# Evaluation of the Patient Experience with the Mawid App during the COVID-19 Pandemic in Al Hassa, Saudi Arabia

**DOI:** 10.3390/healthcare10061008

**Published:** 2022-05-30

**Authors:** Eman AlAli, Reem AL-Dossary, Saja Al-Rayes, Naof Al-Ansary, Deema Alshawan, Salma Almulla, Fahad Alanezi, Zahraa Alakrawi, Norah Alnaim, Linah Saraireh, Razaz Waheeb Attar, Nouf Alaenzi, Hayathem bin Hasher, Bashair AlThani, Lojain Alsulaiman, Naif Alenazi, Beyan Hariri, Turki Alanzi

**Affiliations:** 1 College of Public Health, Imam Abdulrahman Bin Faisal University, Dammam 34212, Saudi Arabia; 2220500146@iau.edu.sa (E.A.); salrayes@iau.edu.sa (S.A.-R.); nalansary@iau.edu.sa (N.A.-A.); dshawan@iau.edu.sa (D.A.); smulla@iau.edu.sa (S.A.); zalakrawi@iau.edu.sa (Z.A.); bahariri@iau.edu.sa (B.H.); 2 Nursing Education Department, College of Nursing, Imam Abdulrahman Bin Faisal University, Dammam 34221, Saudi Arabia; rnaldosari@iau.edu.sa; 3 College of Business Administration, Imam Abdulrahman Bin Faisal University, Dammam 34212, Saudi Arabia; fmoalanezi@iau.edu.sa (F.A.); lmsaraireh@iau.edu.sa (L.S.); bfalthani@iau.edu.sa (B.A.); 4 Department of Computer Science, College of Sciences and Humanities in Jubail, Imam Abdulrahman bin Faisal University, Dammam 34212, Saudi Arabia; nmalnaim@iau.edu.sa; 5 Princess Nourah Bint Abdulrahman University, Riyadh 11564, Saudi Arabia; raattar@pnu.edu.sa; 6 Department of Clinical Laboratory Sciences, College of Applied Medical Sciences, Al Qurayyat Jouf University, Sakakah 72388, Saudi Arabia; nouf_nn66@hotmail.com; 7 Healthcare and legal facilities, University of Kent, Kent CT2 7NZ, UK; drhayathem@gmail.com; 8 Qatif central hospital- inherited blood disorders center, 24135, Dammam, Saudi Arabia; ljoon.a22@gmail.com; 9 Diving Unit, Medical Services, Diving and Hyperbaric Medicine Department, 24135, Dammam Saudi Arabia; drnaifalenazi78@gmail.com

**Keywords:** Mawid app, COVID-19 pandemic, mobile application, primary healthcare centers, Al Hassa, Saudi Arabia

## Abstract

(1) Introduction: The objective of this study was to evaluate the patient experience with the Mawid application during the COVID-19 pandemic in Al Hassa, Saudi Arabia. (2) Methodology: A quantitative cross-sectional survey was designed to evaluate the patient experience with the Mawid app during the COVID-19 pandemic in Al Hassa, Saudi Arabia. A total of 146 respondents completed the questionnaire. (3) Results: More than half of the participants (65.8%) opined that application was easy to use. Furthermore, 65.1% of the participants considered it to be very easy and easy to search for the required information; and 63.7% of the respondents reflected that it was easy to book an appointment. There was a statistically significant difference between the ease of searching for the required information (*p*-value = 0.006); the ease of undoing an unwanted move and gender (*p*-value = 0.049); the ease of searching for the required information and educational level (*p*-value = 0.048); the ease of booking an appointment and educational level (*p* = 0.049); and the ease of searching for the required information and the labor sector of the respondents (*p* value= 0.049) among the genders. No significant differences were identified among the age groups. (4) Conclusions: Overall, most participants suggested that the Mawid app was easy to use and had a potentially useful set of features to help mitigate and manage the COVID-19 pandemic in Al Hassa, Saudi Arabia.

## 1. Introduction

The COVID-19 pandemic that emerged at the end of 2019 in the city of Wuhan, China, has caused an impact on all aspects of the daily life of human beings on a global scale [1]. According to the World Health Organization, as of 25 February 2022, there have been 430,257,564 confirmed cases of COVID-19, including 5,922,047 deaths [2].

Faced with this situation, all of the countries of the world have made multiple efforts to mitigate the disease and stop the spread of the virus [3]. Several efforts have been directed at producing medicines and vaccines, and developing technological systems, such as telemedicine, to provide healthcare services based on advances in information and communication technologies [4].

In this regard, some countries of the world have developed healthcare informatics applications to notify people about the levels of the spread of the COVID-19 virus, report cases of COVID-19, raise awareness about COVID-19, book appointments, disseminate preventive measures on a massive scale, carry out tests to detect the COVID-19 virus, give information to people on available healthcare sites, self-manage symptoms of the disease, perform remote consultations, etc. [3,4,5].

Several applications have been implemented in various countries of the world, such as Italy, Greece, Singapore, Switzerland, Malaysia, Vietnam, the United Kingdom, Germany, New Zealand, Spain, the USA, Norway, Canada, Brazil, Czech Republic, Austria, France, Bangladesh, Germany, the United Arab Emirates, Bahrain, Oman, Saudi Arabia, Kuwait, Hungary, Israel, China, India, Ghana, Japan, Iceland, and Australia, among others [3,6,7,8,9,10]. Many of these applications are based on GPS and Bluetooth technologies and focus on the use of contact tracing apps, digital consultation, appointments booking, etc. [11]. These applications have incorporated functionalities capable of consultation support, self-evaluation, and other features. None of the analyzed applications have incorporated social media platforms [11].

It is pertinent to mention that there are many concerns related to patient privacy, data security and protection, and technical barriers to using these applications [12,13,14,15,16]. These privacy concerns decrease people’s willingness to use these applications [13]. Similarly, a recent study [17] has identified 39 factors from 15 studies for evaluating the eHealth application from multiple stakeholders’ perspective. Few factors that can be related to eHealth users include computer literacy levels, knowledge about eHealth, cost-effectiveness of application, cultural constraints, willingness to use, resistance to change, privacy and security, etc. Moreover, studies [17,18,19,20] have adopted different factors and models, such as perceived usefulness, technology adoption models, etc., for evaluating the acceptance of eHealth applications. These factors were derived from the studies focused on different applications, reflecting that every application can have few general acceptance factors, and there can be few factors in specific to each application that can be analyzed based on its use/purpose of development. Furthermore, some studies have suggested that for an application to be effective in controlling the COVID-19 pandemic, it must have broad public support and must be used by more than 50% of the population [12,21]. This situation suggests that efforts are needed to encourage people to use these apps to slow down the spread of the COVID-19 virus and mitigate the impact of this disease [13].

Regarding Saudi Arabia, the healthcare system has been adapted and changed to handle the evolution of the COVID-19 pandemic. In this regard, during the pandemic, the digital healthcare areas have been strengthened; technologies associated with telemedicine, artificial intelligence, machine learning, and computer networks were developed to increase awareness and knowledge about the COVID-19 pandemic [21].

In addition, the Saudi Arabian health authorities developed three new applications: Tetamman, Tawakkalna, and Tabaud [21]. Tetamman was designed to provide COVID-19 test results, to ask for help, to check up on COVID-19 symptoms, to contact positive cases, and provide alerts through messages. Tawakkalna was developed to request permits to travel during the curfew and report suspected cases of COVID-19 infected people. In addition, Tabaud was created for people to notify if they had previous contact with people infected with COVID-19, and request medical support (50) [3]. Moreover, several applications developed before the pandemic, such as HESN, Mawid, SEHA, and Sehaty, were modified and adapted to handle certain aspects of the COVID-19 pandemic.

Studies have evaluated other mHealth applications in Saudi Arabia. For instance, Tawakkalna application was identified to be average in terms of users experience and efficiency [22]. Another study evaluated Seha mHealth application [23] and identified that it was effective in delivering healthcare services by improving access through streamlined appointment bookings. Similarly, Mawid application was also identified to be easy to use and the users were highly satisfied with the services provided by the application [24]. Furthermore, another study reviewed 12 mHealth applications in Saudi Arabia [9] and identified that different applications were developed for different purposes, such as contact tracing, awareness building, appointment booking, online consultation, etc.; therefore, analyzing the experiences using common scales may not deliver effective outcomes.

For example, Sehhaty was adapted to book appointments for COVID-19. SEHA was reformed to provide information about COVID-19 awareness. HESH was modified to include records for COVID-19 patients. The Mawid application, related to the objective of the present investigation, was improved during the pandemic to allow people to book, cancel, and change or schedule new appointments at 2400 healthcare primary centers located in Saudi Arabia. It can also provide information on COVID-19 awareness and can be used to trace COVID-19 patients [3,25]. In addition, the application can provide patients with information on the precautions that should be taken to avoid becoming infected with the COVID-19 virus in case of travel and other events [25].

**Figure 1:**
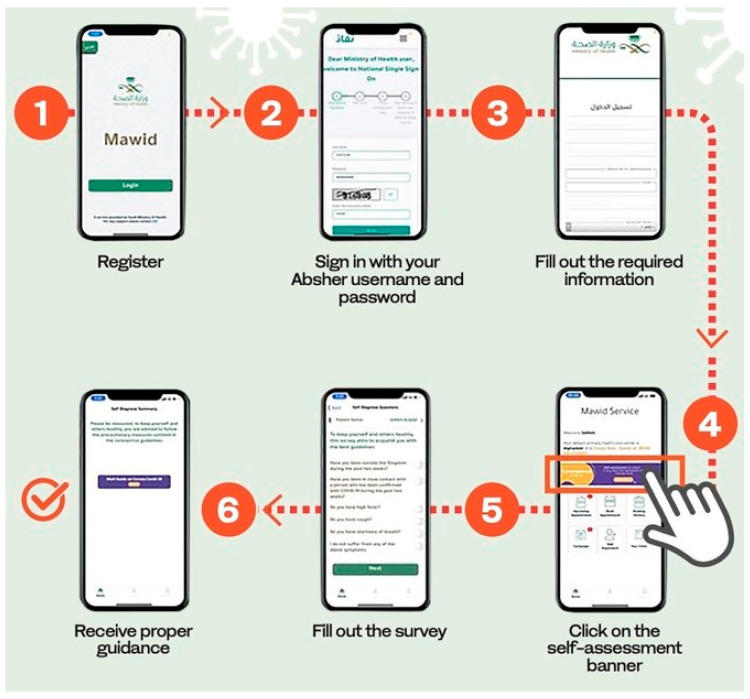
COVID-19 self-assessment test in Mawid application.

Mawid is a mobile application provided by the Ministry of Health to enable patients to book, cancel and/or reschedule their appointments at primary healthcare centers, as well as manage their referral appointments [26]. It helps the users to assess the risk of COVID-19 contamination. Users are advised to enter symptoms and their travel details into the application for the risk assessment test. It also helps users in increasing awareness about COVID-19, and the precautionary measures to be taken, in addition to booking, cancelling and/or rescheduling appointments [25,27]. The assessment process using the application is presented in Figure 1.

Users can book appointments at 2400 healthcare centers across Saudi Arabia, free of cost, using the application. In addition, the application has provided >500,000 consultations regarding COVID-19 and >250,000 self-assessment tests have been taken by the users [27]. Thus, the application can be an effective tool for not only delivering healthcare services, but also for tracking and monitoring epidemics. Considering its growing use, the application is evaluated in terms of its usability, user satisfaction, user experience, and usefulness.

Although several studies have been carried out in Saudi Arabia on the applications implemented during the COVID-19 pandemic by the health authorities of this country, it is necessary to know the opinion that patients have about the ease of use of these applications to adapt them as best as possible to the demands of the users [3,11,21,25]. In this sense, the objective of this study was to evaluate the patient experience with the MAWID application during the COVID-19 pandemic in Al Hassa, Saudi Arabia.

## 2. Methodology

### 2.1. Study Settings and Participants

A quantitative cross-sectional survey was designed to evaluate the patient experience with the MAWID application during the COVID-19 pandemic in Al Hassa, Saudi Arabia. The questionnaire was developed using Google surveys, for which a survey link was generated, which is distributed to the participants through social media platforms (Facebook, Instagram, Twitter (San Francisco, CA, USA)). Community pages and groups related to Smart wearables were identified, and the survey link was posted in the pages and groups requesting the members to participate in the survey Participants were asked to complete the questionnaires and submit them electronically using the survey link. A total of 146 respondents completed the questionnaire. Ethical approval was granted by the Ethical Review Committees at the Imam Abdulrahman bin Faisal University.

### 2.2. Inclusion and Exclusion Criteria

This study included adult patients who had accessed the Mawid application at least once and were citizens of Al Hassa, Saudi Arabia. The rest of the people from Saudi Arabia were excluded.

### 2.3. Questionnaire Design

**Figure A1:**
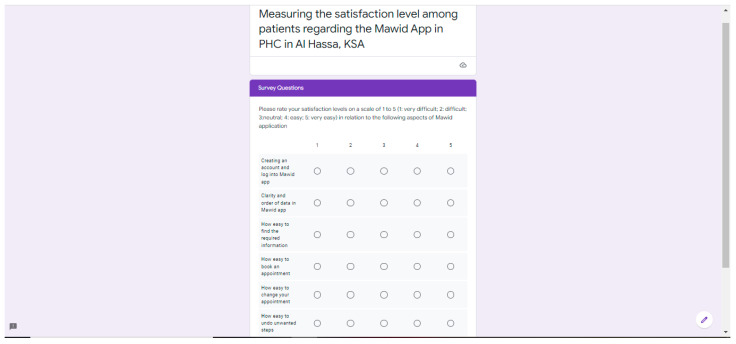
Screenshot of the survey questionnaire.

The questionnaire designed by the research team, and it consisted of two sections. The first section contained five questions related to the demographic information of participants (age, gender, marital status, education level, and profession). The second section included five questions about the ease of use of the MAWID app by patients during the COVID-19 pandemic in Al Hassa, Saudi Arabia (ease of logging into the program, clarity of the data and its arrangement within the program, ease of searching for the required information, ease to reserve an appointment, ease to undo an unwanted move, and ease of changing an appointment). These questions were evaluated using a 5-point Likert scale: very difficult (1), difficult (2), neutral (3), easy (4), and very easy (5). The questionnaire is shown in Appendix A (Table A1), with screenshot of electronic survey (Figure A1).

### 2.4. Sampling Method

A snowball random sampling technique was used to recruit the participants. The sample size included the total number of patients who completed and submitted the survey electronically. Furthermore, the participants were requested to forward the survey link to their friends and colleagues so that larger number of responses can be received.

### 2.5. Data Collection

The data were collected during 1 October 2021 to 31 October 2021. The purpose and significance of the study were explained to the respondents. They were informed that their participation was voluntary, and their responses will be confidential. A summary of the project and the researchers’ contact details were provided to the participants. Participants were informed that answering and submitting the survey indicated that they consented to participate in the survey.

### 2.6. Validity and Reliability of the Questionnaire

The questionnaire was validated by three expert professors from the Imam Abdulrahman bin Faisal University. Furthermore, to evaluate the reliability of the questionnaire, a pilot study was carried out with five participants. Analysis of pilot study results revealed Cronbach alpha (α) value to be greater than 0.70 for all the relevant items, indicating good reliability and internal consistency [28].

### 2.7. Data Analysis

Data were extracted, revised, coded, and fed into IBM SPSS version 22 (Armank, NY, USA). The statistical analysis was performed using two-tailed tests. *p*-values less than 0.05 were considered statistically significant. For all the variables, a descriptive analysis was performed based on the distribution of frequencies expressed in percentages. The relationships between the categorical variables were tested using the Pearson χ^2^ test.

## 3. Results

The demographic information of the respondents is shown in Table 1. The data indicated that more than half of the participants (80.2%) were below 40 years of age. Furthermore, most of them were female (97.5%) and married (78.1%). In addition, 71.2% of the participants were bachelor’s degree holders, and 42.5% of the respondents worked in the government sector.

**Figure 2:**
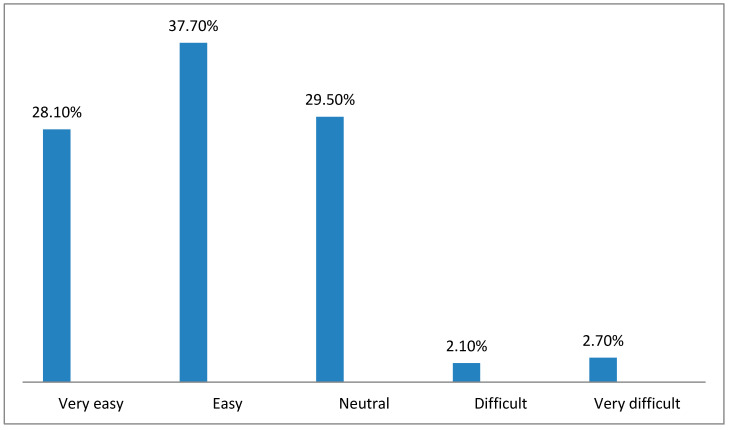
Ease of logging into the program of the Mawid application (*n =* 146).

**Figure 3:**
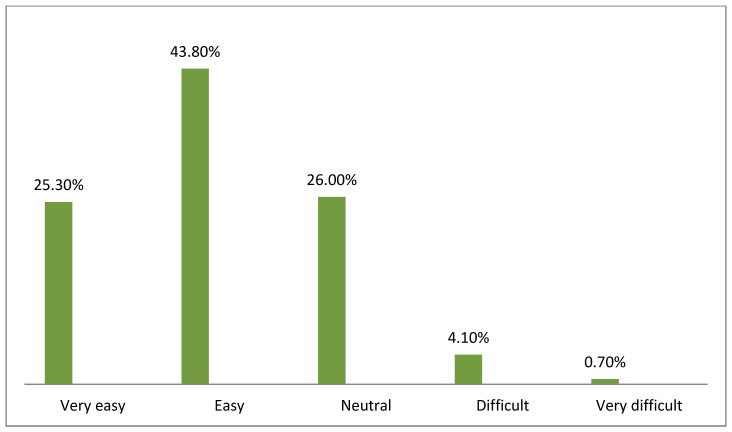
Clarity of the data and its arrangement within the program (*n =* 146).

**Figure 4:**
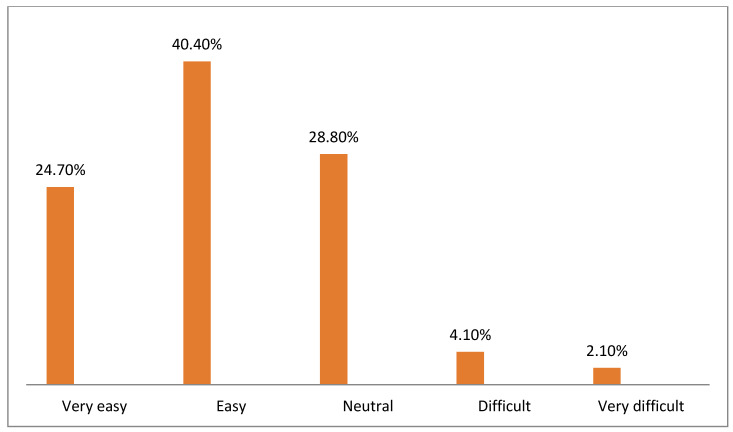
Ease of searching for the required information (*n =* 146).

**Figure 5:**
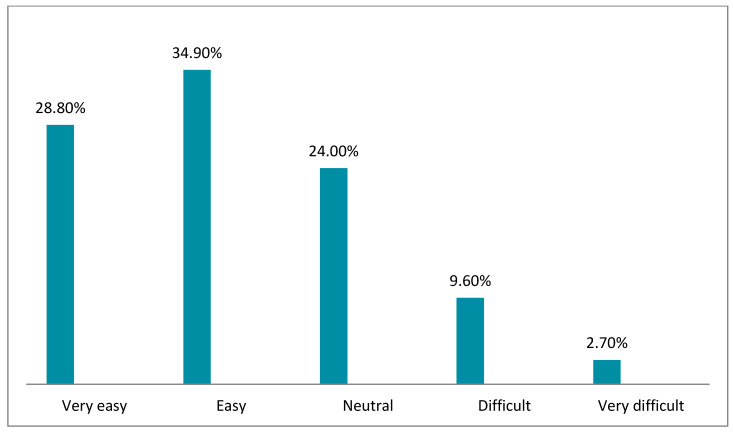
Ease to book an appointment (*n =* 146).

**Figure 6:**
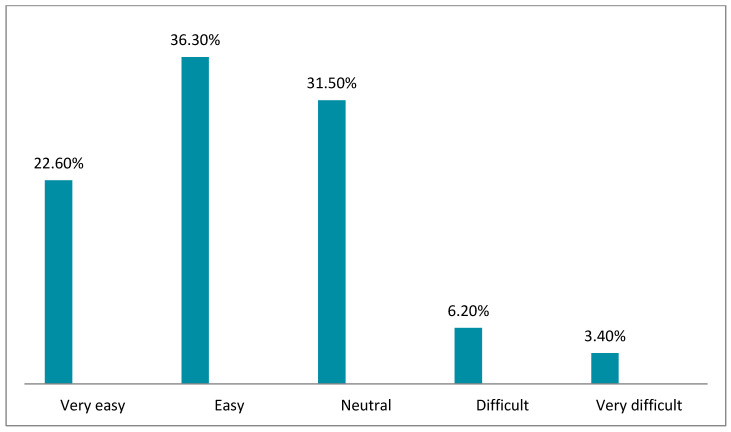
Ease to change an appointment (*n =* 146).

**Figure 7:**
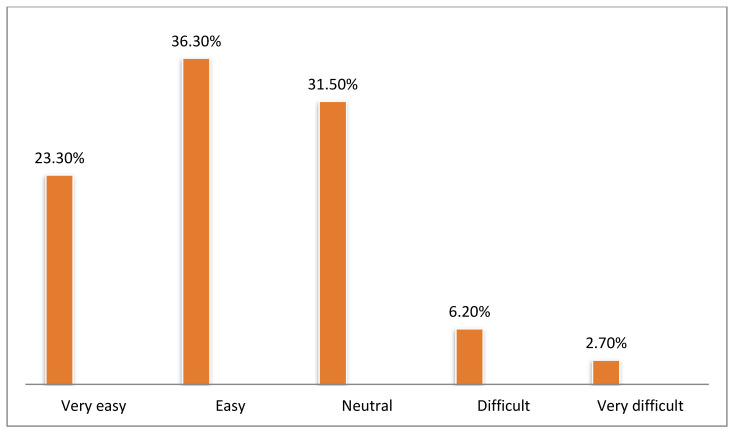
Ease to undo an unwanted movement (*n =* 146).

Figure 2, Figure 3, Figure 4, Figure 5, Figure 6 and Figure 7 illustrates the opinion of the respondents (very easy, easy, neutral, difficult, or very difficult) related to the ease of use of the Mawid application (ease of logging into the application, clarity of the data and its arrangement within the program, ease of searching for the required information, ease to reserve an appointment, ease to undo an unwanted move, and ease to change an appointment).

Figure 2 shows that more than half of the participants (65.8%) thought that it was easy and very easy to log into the application program.

Figure 3 indicates that 69.1% of the respondents believed that it was easy and very easy to handle the application and believed that the data was adequately arranged in the application program.

Figure 4 displays that 65.1% of the participants considered that it was very easy and easy to search for the required information.

Figure 5 indicates that 63.7% of the respondents reflected that it was easy and very easy to book an appointment.

Figure 6 shows that 58.9% of the participants expressed that it was easy and very easy to change an appointment.

Finally, Figure 7 suggests that more than half of the participants (59.6%) expressed that it was easy and very easy to undo unwanted movements.

On the other hand, Table 2, Table 3, Table 4 and Table 5 shows the statistical relationship between the ease of use of the Mawid app (ease of logging into the application, clarity of the data and its arrangement within the program, ease of searching for the required information, ease to reserve an appointment, ease to undo an unwanted move, and ease to change an appointment) and age, gender, educational level, and work location of the participants.

Table 2 suggests that there was not a significant statistical relationship between the ease of use of the Mawid app and age.

Table 3 indicates that there was a significant statistical difference between the ease of searching for the required information and gender (*p*-value = 0.006), and between the ease to undo an unwanted move and gender (*p*-value = 0.49).

Table 4 describes that there was a significant statistical difference between the ease of searching for the required information and educational level (*p*-value = 0.048), and between the ease to reserve an appointment and educational level (*p* = 0.049).

Table 5 suggests that there was a significant statistical difference between the ease of searching for the required information and the working sector of respondents (*p* value = 0.049).

## 4. Discussion

The findings of this study on the evaluation of the patient experience with the Mawid application during the COVID-19 pandemic in Al Hassa, Saudi Arabia, suggested that more than half of the respondents thought that it was easy and very easy to log into the application and that the data was adequately arranged in the app program. Moreover, these participants believed that it was easy and very easy to search for the required information, to reserve an appointment, undo an unwanted move, and change an appointment. Less than a third of the participants had a neutral opinion regarding the mentioned attributes of the application. The rest of the participants, about a tenth of them, believed that it was difficult and very difficult to manage the features of the application. It is important to mention that since the COVID-19 outbreak in Saudi Arabia, the application has delivered consultation services for more than half a million people [27,29].

Similarly, a previous study conducted in Saudi Arabia showed that more than 50% of the participants who had accessed the Mawid app at least once considered it had a good interface to find the information on COVID-19 available in Saudi Arabian healthcare centers [24]. This interface facilitated the search for medical appointments, remote communication between patients and doctors, and knowledge of the symptoms and treatment of COVID-19 [24]. Furthermore, according to the participants, most of the instructions of the Mawid app were easy to follow. In general, people and Saudi Arabian government health authorities have found that the Mawid app was easy to use [27,29,30].

In a different context, a study conducted in the Netherlands revealed that participants positively valued the information available in an app designed to offer education, self-assessment, and monitoring of the COVID-19 [31]. Another study conducted in the United States found that the MyCOVIDKey app was a useful tool for COVID-19 contact tracing, but it needed simple modifications to improve usability [32]. Similarly, in a study carried out in England, participants viewed the NHS COVID-19 app positively [33]. However, the respondents considered that the interface was challenging, difficult, and complex. They thought that these factors would limit its use by the UK population (79) [33].

On the other hand, there was a statistically significant difference between the ease of searching for the required information and gender, the ease of undoing an unwanted move and gender, the ease of searching for the required information and educational level, the ease of booking an appointment and educational level, and the ease of searching for the required information and the labor sector of the respondents. However, there was no statistically significant relationship between the ease of use of the Mawid app and the rest of the variables. A preceding study also revealed that there were significant statistical differences between the ease of use and age, and between the ease of use and gender [24].

The main limitation of this study was the small sample size, which limits the generalizability of the results on the ease of use of the Mawid app by COVID-19 patients in Al Hassa, Saudi Arabia. In addition, this study only considered Mawid application in specific, while there are also other applications being introduced by MoH, Saudi Arabia. Moreover, this study was conducted in single setting (Al Hassa city), even though Mawid application is used across Saudi Arabia. Future studies should aim to increase the sample size of participants, including other respondents from different cities in Saudi Arabia. It would also be interesting to assess the level of acceptance of the applications used during the COVID-19 pandemic in Saudi Arabia.

Despite limitations, this study has both theoretical and practical implications. Firstly, the study contributes to the literature relating to mHealth applications in the context of Middle East. Secondly, the findings from this study can aid decision-makers such as Ministry of Health in improving the Mawid or other similar applications according to the attitudes of the users (findings from this study). Furthermore, the findings can also be generalized in the Middle east context where similar applications are developed and implemented.

## 5. Conclusions

Overall, most participants suggested that the Mawid app was easy to use and had a potentially useful set of features to help mitigate and manage the COVID-19 pandemic in Al Hassa, Saudi Arabia. In addition, there was a statistically significant difference between the ease of searching for the required information and gender, the ease of undoing an unwanted move and gender, the ease of searching for the required information and educational level, the ease of booking an appointment and educational level, and the ease of searching for the required information and the labor sector of the respondents.

## Figures and Tables

**Table 1:** Demographic information (*n =* 146).

	*n*	%
Age (years)		
18–30	63	43.2%
31–40	54	37.0%
>40	29	19.9%
Gender		
Male	11	7.5%
Female	135	92.5%
Marital status		
Single	23	15.8%
Married	114	78.1%
Divorced/widow	9	6.2%
Education level		
High school student	29	19.9%
Diploma	13	8.9%
Bachelor	104	71.2%
Work sector		
Student	50	34.2%
Governmental employee	62	42.5%
Privet sector employee	34	23.3%

**Table 2:** Statistical relationship between the ease of use of the Mawid app and age (*n =* 146).

	Age						*p*-Value
	18–30		31–40		>40		
	*n*	%	*n*	%	*n*	%	
Ease of logging into the program							0.422
Easy	42	66.7%	33	61.1%	21	72.4%	
Neutral	17	27.0%	20	37.0%	6	20.7%	
Difficult	4	6.3%	1	1.9%	2	6.9%	
Clarity of the data and its arrangement within the program							0.614
Easy	44	69.8%	37	68.5%	20	69.0%	
Neutral	18	28.6%	13	24.1%	7	24.1%	
Difficult	1	1.6%	4	7.4%	2	6.9%	
Ease of searching for the required information							0.830
Easy	41	65.1%	33	61.1%	21	72.4%	
Neutral	18	28.6%	18	33.3%	6	20.7%	
Difficult	4	6.3%	3	5.6%	2	6.9%	
Ease to reserve an appointment							0.901
Easy	43	68.3%	33	61.1%	17	58.6%	
Neutral	13	20.6%	14	25.9%	8	27.6%	
Difficult	7	11.1%	7	13.0%	4	13.8%	
Ease to undo an unwanted move							0.352
Easy	36	57.1%	34	63.0%	17	58.6%	
Neutral	18	28.6%	17	31.5%	11	37.9%	
Difficult	9	14.3%	3	5.6%	1	3.4%	
Ease to change an appointment							0.665
Easy	38	60.3%	28	51.9%	20	69.0%	
Neutral	19	30.2%	20	37.0%	7	24.1%	
Difficult	6	9.5%	6	11.1%	2	6.9%	

**Table 3:** Statistical relationship between the ease of use of the Mawid app and gender (*n =* 146).

	Gender				*p*-Value
	Male		Female		
	*n*	%	*n*	%	
Ease of logging into the program					0.785
Easy	7	63.6%	89	65.9%	
Neutral	3	27.3%	40	29.6%	
Difficult	1	9.1%	6	4.4%	
Clarity of the data and its arrangement within the program					0.772
Easy	7	63.6%	94	69.6%	
Neutral	3	27.3%	35	25.9%	
Difficult	1	9.1%	6	4.4%	
Ease of searching for the required information					0.006 *
Easy	7	63.6%	88	65.2%	
Neutral	1	9.1%	41	30.4%	
Difficult	3	27.3%	6	4.4%	
Ease to reserve an appointment					0.601
Easy	6	54.5%	87	64.4%	
Neutral	4	36.4%	31	23.0%	
Difficult	1	9.1%	17	12.6%	
Ease to undo an unwanted move					0.049 *
Easy	8	72.7%	79	58.5%	
Neutral	1	9.1%	45	33.3%	
Difficult	2	18.2%	11	8.1%	
Ease to change an appointment					0.600
Easy	6	54.5%	80	59.3%	
Neutral	3	27.3%	43	31.9%	
Difficult	2	18.2%	12	8.9%	

* Statistically significant difference.

**Table 4:** Statistical relationship between the ease of use of the Mawid app and educational level (*n =* 146).

	Educational Level						*p*-Value
	High School Student		Diploma		Bachelor		
	*n*	%	*n*	%	*n*	%	
Ease of logging into the program							0.190
Easy	19	65.5%	6	46.2%	71	68.3%	
Neutral	10	34.5%	5	38.5%	28	26.9%	
Difficult	0	0.0%	2	15.4%	5	4.8%	
Clarity of the data and its arrangement within the program							0.241
Easy	20	69.0%	7	53.8%	74	71.2%	
Neutral	9	31.0%	4	30.8%	25	24.0%	
Difficult	0	0.0%	2	15.4%	5	4.8%	
Ease of searching for the required information							0.048 *
Easy	20	69.0%	8	61.5%	67	64.4%	
Neutral	9	31.0%	2	15.4%	31	29.8%	
Difficult	0	0.0%	3	23.1%	6	5.8%	
Ease to reserve an appointment							0.049 *
Easy	18	62.1%	5	38.5%	70	67.3%	
Neutral	6	20.7%	4	30.8%	25	24.0%	
Difficult	5	17.2%	4	30.8%	9	8.7%	
Ease to undo an unwanted move							0.472
Easy	20	69.0%	5	38.5%	62	59.6%	
Neutral	7	24.1%	6	46.2%	33	31.7%	
Difficult	2	6.9%	2	15.4%	9	8.7%	
Ease to change an appointment							0.375
Easy	18	62.1%	5	38.5%	63	60.6%	
Neutral	8	27.6%	5	38.5%	33	31.7%	
Difficult	3	10.3%	3	23.1%	8	7.7%	

* Statistically significant difference.

**Table 5:** Statistical relationship between the ease of use of the Mawid app and working sector (*n =* 146).

	Job						*p*-Value
	Student		Governmental Employee		Private Sector Employee		
	*n*	%	*n*	%	*n*	%	
Ease of logging into the program							0.320
Easy	36	72.0%	38	61.3%	22	64.7%	
Neutral	14	28.0%	20	32.3%	9	26.5%	
Difficult	0	0.0%	4	6.5%	3	8.8%	
Clarity of the data and its arrangement within the program							0.233
Easy	38	76.0%	39	62.9%	24	70.6%	
Neutral	12	24.0%	19	30.6%	7	20.6%	
Difficult	0	0.0%	4	6.5%	3	8.8%	
Ease of searching for the required information							0.049 *
Easy	34	68.0%	38	61.3%	23	67.6%	
Neutral	16	32.0%	17	27.4%	9	26.5%	
Difficult	0	0.0%	7	11.3%	2	5.9%	
Ease to reserve an appointment							0.247
Easy	36	72.0%	33	53.2%	24	70.6%	
Neutral	9	18.0%	20	32.3%	6	17.6%	
Difficult	5	10.0%	9	14.5%	4	11.8%	
Ease to undo an unwanted move							0.530
Easy	31	62.0%	34	54.8%	22	64.7%	
Neutral	13	26.0%	24	38.7%	9	26.5%	
Difficult	6	12.0%	4	6.5%	3	8.8%	
Ease to change an appointment							0.504
Easy	34	68.0%	33	53.2%	19	55.9%	
Neutral	11	22.0%	23	37.1%	12	35.3%	
Difficult	5	10.0%	6	9.7%	3	8.8%	

* Statistically significant difference.

## Data Availability

The data presented in this study are available on request from the corresponding author. The data are not publicly available due to privacy clause.

## Appendix A

### Appendix A.1. Survey Questionnaire

#### Measuring the Satisfaction Level among Patients Regarding the Mawid App in PHC in Al Hassa, KSA

Greetings,

Your participation and response to this survey will help us achieve our goal as postgraduate students in healthcare quality and patient safety at Imam Abdulrahman Bin Faisal University and contribute to the study of the customer’s satisfaction about the use of the Mawid app.

Targeted group:

- The customers of healthcare centers in Al Hassa, Saudi Arabia
- Male/female.
- Age: 18 years and above.

The purpose of this study:

Through this study, we seek to measure the satisfaction level of the customers about the ease of use of the Mawid app in Al Hassa region in Saudi Arabia. The main objective is to evaluate the patient experience with the MAWID application during the COVID-19 pandemic in Al Hassa, Saudi Arabia.

Note:

- The Questionnaire takes just a few minutes to complete
- Please note that the Questionnaire contains 12 questions
- Your participation in the survey is voluntary. You can withdraw by cancellation at any time, and will not have any repercussions. We will not request any special or sensitive information such as names. Also, the information obtained will remain anonymous and will be stored and processed confidentially. There are no risks associated with this survey as you will not be contacted in person. No names, contact information, or biological samples will be obtained from the participants.

I hope you fill out the following Questionnaire:

Please answer the following questions:

1. Measuring the level of satisfaction with the Mawid program

**Table A1:** Measuring the level of satisfaction with the Mawid program.

Items	Very Difficult	Difficult	Neutral	Easy	Very Easy
Creating an account and log into Mawid app	1	2	3	4	5
Clarity and order of data in Mawid app	1	2	3	4	5
How easy to find the required information	1	2	3	4	5
How easy to book an appointment	1	2	3	4	5
How easy to change your appointment	1	2	3	4	5
How easy to undo unwanted steps	1	2	3	4	5

2. Demographic information:
  ∗ Age: (18–30/31–40/>41)
  ∗ Gender: (male/female)
  ∗ Social status (single/married)
  ∗ Academic level (secondary/diploma/bachelor’s/postgraduate)
  ∗ Profession: (student/government employee/private sector employee)
3. Would you like to add notes?

Thank you
